# Comparing Different Classifiers in Sensory Motor Brain Computer Interfaces

**DOI:** 10.1371/journal.pone.0129435

**Published:** 2015-06-19

**Authors:** Hossein Bashashati, Rabab K. Ward, Gary E. Birch, Ali Bashashati

**Affiliations:** 1 Electrical and Computer Engineering Department, University of British Columbia, Vancouver, BC, Canada; 2 Department of Molecular Oncology, British Columbia Cancer Agency, Vancouver, BC, Canada; 3 Neil Squire Society, Burnaby, BC, Canada; University of Electronic Science and Technology of China, CHINA

## Abstract

A problem that impedes the progress in Brain-Computer Interface (BCI) research is the difficulty in reproducing the results of different papers. Comparing different algorithms at present is very difficult. Some improvements have been made by the use of standard datasets to evaluate different algorithms. However, the lack of a comparison framework still exists. In this paper, we construct a new general comparison framework to compare different algorithms on several standard datasets. All these datasets correspond to sensory motor BCIs, and are obtained from 21 subjects during their operation of synchronous BCIs and 8 subjects using self-paced BCIs. Other researchers can use our framework to compare their own algorithms on their own datasets. We have compared the performance of different popular classification algorithms over these 29 subjects and performed statistical tests to validate our results. Our findings suggest that, for a given subject, the choice of the classifier for a BCI system depends on the feature extraction method used in that BCI system. This is in contrary to most of publications in the field that have used Linear Discriminant Analysis (LDA) as the classifier of choice for BCI systems.

## Introduction

A Brain Computer Interface is a system that discovers specific patterns in a person′s brain activity that relate to the his/her intention to control a device [1]. If such patterns are detected in the EEG, then the BCI issues specific signals to put into effect the intended action. Traditionally, the most popular application of BCIs has been to assist disabled people [2], however, new applications such as playing computer games have emerged recently [3].

Among the various means to measure the brain activity, the EEG-based BCI systems have several advantages [4]. They can measure the changes in the brain activity over short periods of time (milliseconds), they are inexpensive, non-invasive and versatile. However, they also have several drawbacks. The signal to noise ratio of the EEG signal is low, i.e., the signals have very low amplitude (i.e. about 10 to 100 micro volts) compared to the background noise. Therefore, detecting the intentions of a person from his measured brain signals is a challenging task and has been at the forefront of research. Furthermore, the resolution of brain signals referred to as the spatial resolution is low and dependent on the number of electrodes that could be placed on the subject’s head.

Present-day BCIs use several different electrophysiological activities to operate. Among the various different methods to operate a BCI, those that rely on the sensory motor have been of special interest [4][5]. This kind of BCIs does not need any stimuli from outside the BCI to control the system and the subject can learn to generate the appropriate brain pattern to control a device voluntarily. The focus of this paper is on sensory motor BCI systems.

When a person performs a motor activity such as limb movement, changes that are frequency specific would result in the EEG activity. Generally such changes may result in decrease or increase in the power of certain frequency bands in the EEG signals [6]. This may be considered due to a decrease or an increase in the synchrony of the underlying neuronal populations. The decrease in the synchrony is called Event Related Desynchronization (ERD), and the increase is referred to as Event Related Synchronization (ERS). It has been shown that cortical activation that is related to movement preparation and execution desynchronizes the upper alpha and the lower beta rhythms [6]. It has also been shown that a motor imagery activity (i.e., an imagined movement) generates patterns in the EEG that are similar to those generated by actual movements [7]. BCIs that are motor imagery based are especially suitable for disabled people who do not have control over their limbs.

Sensory motor (and other) BCI systems can be categorized by two different paradigms, namely synchronous and self-paced systems [8][9]. The majority of the research in BCIs have concentrated on synchronous systems. In synchronous BCIs, the subjects can only control the BCI output during system-defined periods and therefore, they cannot control the output in other times. On the other hand, in self-paced BCIs, the users have the option of controlling the system whenever they intend to do so. The periods during which the user is not controlling the system are called the No-Control (NC) states. The system will not issue any control signal during the NC states. Despite the significant improvements in self-paced BCIs, compared to synchronous BCI systems, designing self-paced BCIs remains extremely challenging.

Even though much interest and progress have been made during the past two decades in BCI research, this field is still at its infancy. Many improvements are still necessary in order to encourage its widespread adoption. Current BCI systems are not accurate enough and are far from perfect to operate in online settings. Particularly for noninvasive BCIs, which are by far the most widely used BCIs for measuring the brain activity, some characteristics of the recorded signal (e.g. the low signal to noise ratio, susceptibility to artifacts, etc.) make it challenging to extract people′s intentions from their brain waves. Therefore, the accuracy of BCI systems has not yet reached required high level to enable them in day-to-day human life applications, this is especially true for self-paced BCIs, which are ultimately the more natural way to operate BCIs [10].

Regarding the signal processing aspects of BCI research, feature extraction and classification have been studied separately. However, a more optimal learning framework for a BCI problem should aim at studying feature extraction and classification jointly, considering the fact that the performance of the classifier depends on the choice of the feature extraction method. In other words, we should customize the classifier and feature extractor based on each other. For instance, selecting a particular feature extraction method might cause the data samples of different brain states to be linearly separable in the feature space. As a result, selecting a linear classifier would be a better choice for discriminating between the brain states.

Most BCI systems utilize the Linear Discriminant Analysis (LDA) classifier for classification purposes [5][11]. The LDA classifier has a very strong assumption that the conditional probability densities of the two classes have a Gaussian distribution with the same covariance function. As a result, the discriminant function is linear and may not be suitable for non-linearly separable feature spaces. In addition, this classifier is very sensitive to outliers. On the other hand, the main challenges in classification of EEG data in BCIs is that the data is non-stationary, noisy, contains outliers, usually of high dimensions and the training set for learning the patterns of the data is unfortunately small [12][13]. As a result, the classification strategies in BCI systems should be able to cope with these problems appropriately.

Due to the variations in BCI technologies and the lack of a general comparison framework in BCIs, it is almost infeasible to compare existing BCI technologies and consequently the progress in the field is slow. This is due to the fact that most of scientific papers have not described the experimental details which are important for replication of the results. Also, the data used in several papers are not shared. There are some general purpose software systems [14][15] that facilitate the research in BCIs. However, these systems are mostly suitable for data acquisition and for real time processing of brain signals. The purpose of BCI competitions was to encourage scientists to apply their algorithms on the same data. Besides sharing the data, it is also essential to share the source code so as to enable the results to be reproduced and move the field forward at a faster pace. The idea of reproducibility and data sharing has been practiced in other fields and yielded profound improvements in making meaningful progress [16].

In this study, we have built a unified comparison framework to evaluate the performance of different classifiers on several sensory motor BCI datasets. The data used are those from BCI competitions [17][18][19][20], as well as one of our own datasets [21]. There have been some efforts to compare the performance of different classifiers in BCI systems [22][23][24][25][26]. However, these comparisons have been performed for a small number of subjects on small number of classification methods. In [27], the authors surveyed the performance of different classifiers in BCIs, however, the best way to compare the classifiers is to evaluate their performance in the same context, i.e., on the same set of subjects using the same set of features with the same set of parameters.

This study provides an open source comparison framework for BCI systems and is unique in three aspects. First, the number of subjects considered in this paper are 21 subjects using synchronous BCIs and 8 subjects using self-paced BCIs (i.e. an overall of 29 subjects). Other studies have been performed on smaller sample sizes (less than 5 subjects) and as a result, their findings may not be generalized in a larger subject pool. Secondly, we have used statistical tests to compare the performance of different algorithms. This will enable us to examine whether or not there is significant differences between the performance of different classifiers. Other studies have not employed statistical tests for comparisons, mainly due to their smaller sample sizes and have solely relied on the mean performance of the classifiers (which is not a robust measure), as a surrogate for the overall performance of a specific classifier. Thirdly, to increase the transparency, the source code of our experiments is openly available so that other researchers can apply it on their own data and benchmark their algorithms with standard methods.

In the following sections we first explain the general structure of the comparison framework, then we explain the datasets used and finally the results of the different algorithms are compared.

## Methods

**Fig 1:**
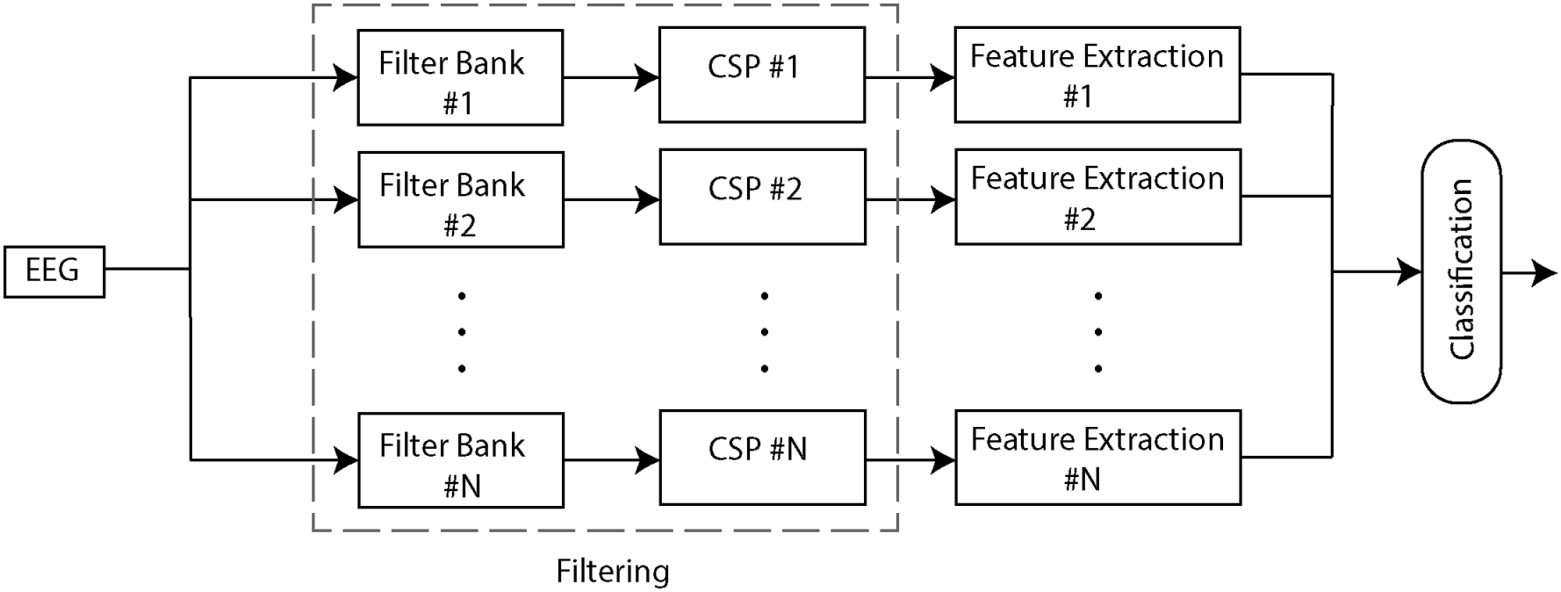
The BCI framework. Our framework has three main steps: 1. Filtering, 2. Feature Extraction and 3. Classification. The output of each step is fed to the next step.

The overall structure of our comparison framework is shown in Fig 1. This framework has three main sequential processing components, Filtering, Feature Extraction and Classification. All data from the available EEG channels are fed as the input, the Filtering component performs frequency filtering and spatial filtering, and the Feature Extraction component extracts features from the filtered data. Finally, in the last step the extracted features are combined to build a dataset which is then fed to the classification component. In the following subsections, the details of each of the above mentioned components are explained.

### Spatial and Frequency Filtering

The first step of our framework is composed of frequency filtering and spatial filtering. Frequency filtering is done using a filter bank. A filter bank is an array of band pass filters and contains n blocks. Each block corresponds to filtering the original signal in a specific sub-band. For frequency filtering, we have used the fifth order Butterworth filter. This filter has a flat frequency response in the pass band. The result of applying each filter-bank block is fed to the next block in which spatial filtering is performed on the signal.

Common Spatial Patterns (CSP) [28][29] is used for spatial filtering. CSP has been widely used in BCI systems and has yielded considerable improvements in the signal to noise ratio of the EEG signal. CSP projects multiple channels of the EEG data onto a surrogate sensor space by applying a linear transformation on the EEG data. This technique is a supervised method of combining multiple channels and has been developed for binary classification. This method maximizes the variance of signals for one class while minimizing the variance of the signals for the other. As the signals are standardized before applying CSP, the variance of the signals would be equivalent to their band power. Thereby, if we use the band power as the extracted feature in the surrogate space, the discrimination of the classes (e.g., right and left hand imagery movements) would be maximized.

Suppose the normalized covariance matrices of both classes for each trial are given by Σ^*c*^ where *c* ∈ {+,−}. The covariance matrices are *N* × *T* where N is the number of channels and T is the number of samples. The CSP algorithm maximizes the following equation:

$$\begin{array}{c} \arg\;\max_{W}\frac{W^{T}\Sigma^{+}W}{W^{T}\Sigma^{-}W} \end{array}$$ (1)

where *W* is the projection matrix. This equation can be solved by applying simultaneous diagonalization of the covariance matrices of both classes. By multiplying the projection matrix by the original EEG signal we can obtain the uncorrelated brain signals.

The benefit of applying CSP is that we can select a subset of filters that preserves as much information as possible and discriminates the two classes very well. However, choosing the number of filters (i.e., spatial patterns) is difficult, and is usually determined by heuristic approaches. CSP is inherently designed for 2-class BCI tasks. To use CSP for multi-class problems, we use a one-against-others scheme.

### Feature Extraction

In the feature extraction step, different kinds of features can be extracted from the filtered signal. The extracted features should properly represent the information hidden in the raw EEG signal. We use two of the most common and successful feature extraction methods utilized in motor-imagery based BCI systems. Both approaches calculate the band-power (BP) of the signal [30][31][32].

In the first approach, the band-power of a signal is extracted by directly applying to a signal, a filter that only allows the frequencies inside each filter band to pass. Assuming a perfect block filter, we can then estimate the power of the filtered signal by summing the squares of the magnitude of the filtered signal. Since the logarithm of band-power features has also been used in the literature [30], we have included both the band-power and its logarithm in our feature set. In this study, we extracted the band power features for the *α* [8–12 Hz] and *β* [16–24 Hz] frequency bands of the brain signals. We refer to these band-power features as the BP features in the remainder of the text.

In the second feature extraction approach, we used the Morlet wavelet to extract the band-power features. This method is one of the most successful feature extraction methods used in sensory motor BCI systems [30]. In this method, the EEG signal is decomposed using the Morlet mother wavelet and the power in each frequency band is calculated. We used the exact configuration as in [31] to extract these features; i.e., for each channel, all the frequency bands are chosen to be in the 4 to 30 Hz range. This results in 26 features for each channel. This setting for extracting BP features will result in high-dimensional feature spaces, especially for datasets with many EEG channels.

After extracting features from each block of the filter bank, the extracted features are combined to build a feature vector, which is fed to the classification component of our framework.

### Classification

In supervised learning, given a set of training samples $D=\{({\underline{x}}_{1},y_{1}),({\underline{x}}_{2},y_{2}),\ldots ,({\underline{x}}_{n},y_{n})\}$, the aim is to find an approximation of the unknown function *g*:*X* → *Y* which has generated the samples. Each ${\underline{x}}_{i}$ is a features vector and *y*_*i*_ is the label of the corresponding feature vector. X is the feature matrix and Y is a vector corresponding to the label of each row (i.e. ${\underline{x}}_{i}$) of X. In probabilistic classification, instead of approximating the function *g*, we find a posterior probability $P(c|\underline{x})$ (where *c* is the predicted class label and $\underline{x}$ is the feature vector), and eventually assign the label to the class with the highest probability. This probability can be calculated using the Bayes rule. $P(c|\underline{x})$ is called the posterior and $P(\underline{x}|c)$ is the class conditional density.

$$\begin{array}{c} P\left(c|\underline{x}\right)=\frac{P\left(\underline{x}|c\right)P\left(c\right)}{P(\underline{x})} \end{array}$$ (2)

#### Gaussian Discriminant Analysis

If we assume the class conditional density to have a Gaussian distribution, the resulting classifier is called the Gaussian Discriminant analysis [33]. In other words, this model fits a Gaussian distribution to the samples of each class. If the covariance matrices of both classes are considered the same, the result will be a Linear Discriminant Analysis (LDA) classifier in which the decision boundary is a linear surface. If we do not assume any constraints on the covariance function, the resulting decision boundary is a quadratic function and the corresponding classifier is called Quadratic Discriminant Analysis.

LDA classifier imposes very strong assumption on the underlying distribution of the data. However, the computation of the discriminative function is very efficient and, therefore, LDA has been popular in the BCI field. It is worth mentioning that various algorithms [34][35][36] have been proposed to address the shortcomings of LDA. These algorithms are more robust and some of them, such as Z-LDA, [34] can handle the case where the covariance of two classes are different.

#### Logistic Regression (LR)

Logistic Regression [37] is a discriminative learning classifier that directly estimates the parameters of the posterior distribution function $P(c|\underline{x})$. This algorithm assumes the distribution $P(c|\underline{x})$ is given by Eq 3.

$$\begin{array}{c} P\left(c=k|\underline{x}\right)=\frac{exp({\underline{w}}_{k}^{T}\underline{x})}{\sum_{j=1}^{K}exp\left({\underline{w}}_{j}^{T}\underline{x}\right)} \end{array}$$ (3)

where ${\underline{w}}_{j}$s are the parameters to estimate and *K* is the number of classes. Then maximum likelihood method is used to directly approximate ${\underline{w}}_{j}s$. As the Hessian matrix for the logistic regression model is positive definite, the error function has a unique minimum. Overfitting can occur in logistic regression when the data is sparse and of high dimensions (which is the case in BCIs). We used *L*_1_ and *L*_2_ regularization jointly to cope with the overfitting problem.

#### Random Forests (RF)

Random Forest [38] is an ensemble learning algorithm that is constructed by combining multiple decision trees at the training stage and produces a result that is the average of the output of individual trees. This powerful learning algorithm injects randomness into each tree in two ways. The first uses bootstrapping to sample from the original dataset (i.e. the algorithm takes N samples with replacement from the original dataset). The second is by selecting a subset of the features to split each node of the tree. Injecting randomness in the process of building Random Forests, makes these classifiers robust and cause them to have a good performance when the data have many outliers, which is the case in BCIs [39]. Another consequence of injecting randomness in random forests is the ability to rank the different features and also to acquire a measure for feature importance.

The seminal paper of Random Forests [40] claims that increasing the number of trees does not cause the random forest to overfit. However, [41] has found that RFs can overfit with noisy datasets. As a consequence, along with other important parameters of RF we have tuned this parameter too (refer to Model Selection Section).

#### Support Vector Machines (SVM)

SVM [42] is a discriminative classification algorithm which is able to find a decision hyper-plane with the maximum distance (margin) to the nearest data points (Support Vectors) of each class. As a result, this method has a high generalization power. The decision function of SVM is fully specified by a subset of the training data points, which leads to a sparse solution for SVM. The cost function of SVM is a convex function that leads to an optimal solution for the optimization task.

The mathematical formulation of SVM gives us the ability to use the kernel trick to map the original finite dimensional space into a destination space with much higher dimensions. The use of a kernel trick is beneficial particularly when the data points cannot be separated with a hyper-plane in the original feature space. This may lead to an easier separation of the data points in the destination space. Even though the data points might be mapped into a very high dimensional or even an infinite space the complexity of computing a kernel matrix can be far smaller. Hence, the SVM algorithm circumvents the curse of dimensionality by relying on the data points only through the kernel. In this study, we have only used SVMs with radial basis function (RBF) kernel. As stated in [43], under some conditions, linear kernel SVM can be approximated with an RBF kernel. SVMs are inherently designed for two-class classification tasks. Many different methods have been proposed to adapt SVMs to multi-class problems [44]. We used a one-against-one scheme to adapt SVMs for multi-class problems.

#### Boosting Algorithm

Boosting [45] is a learning algorithm based on the observation that building a highly accurate rule for classification is a really difficult task. This algorithm works by building many rough rules of thumb. These rules are called weak classifiers. Each of these weak classifiers is trained on a subset of the data points. These weak classifiers are then combined into a single prediction rule which would be much more accurate than individual weak classifiers.

There are many variants of the boosting algorithm. The differences amongst these algorithms stem from two issues. The first is concerned with how to choose the subset of the data points for each weak learning algorithm. The second is related to how to combine the output of a weak classifier into a single prediction rule. In this study, we used a variant of boosting called the Adaboost algorithm. In this algorithm, the subset of data points that are used for training a weak learning are the ones that are misclassified by the previous weak learner. For generating a single prediction rule, Adaboost takes a weighted majority vote of the prediction of the weak learners.

#### Multi Layer Perceptron (MLP)

The last classifier used in this paper is a feed forward neural network [37] with one hidden layer. It is proved that an MLP with enough number of neurons in the hidden layer can approximate any function. Despite the flexibility and capability of this algorithm to approximate any nonlinear function, this algorithm can easily overfit and the cost function to optimize is a non-convex function. The cost function used in this paper is the negative log-likelihood function with both *L*_1_ and *L*_2_ regularization to avoid overfitting.

### Performance Measure

For the synchronous datasets, after the labels of different classes of motor imagery tasks were obtained, the classification accuracy was used as the measure to compare the performance of different algorithms.

For self-paced datasets, the true positive rate (TPR) and the false positive rate (FPR) are the most popular measures in the field. It is common to fix the FPR to a small value (e.g. 1 percent) and compare the performance of TPRs of different methods. However, this method does not exploit all the information given by the output of the classifiers because all the classifiers applied in this research produce probability estimates (i.e. confidence) of a sample belonging to each class. Since, the output of the classifiers is not just the label of each sample, a good performance measure should exploit the added information. The receiver operating characteristic (ROC) [46] curve is the best way to compare these classifiers as it illustrates the performance of classifiers for varied discrimination thresholds. ROC is well-suited for self-paced BCIs because ROC is is suitable in evaluating the performance when we have imbalanced data or unequal misclassifications costs. To compare the performances of the methods, we use the Area Under the ROC Curve (AUC). AUC represents the probability that a randomly selected positive sample gets a higher rank than a randomly chosen negative sample. AUC varies between 0 and 1 and in general a higher AUC is better.

### Model Selection

Model selection consists of finding the appropriate set of parameters for each learning algorithm. We adjusted the value of a set parameters (referred to as “BCI parameters”) consisting of parameters of the sensory motor EEG signal and the classifier-specific parameters using a grid-based approach.

A set of candidate parameters including both the BCI parameters and the classifier parameters was considered for each algorithm, and 5 times 5-fold cross validation was performed to find the best parameters. Then the parameters with the best mean performance were used to train a classifier on the training set and the final classifier was used to evaluate the performance on independent test dataset. The values of all these parameters, i.e. the BCI parameters and the classifier parameters were adjusted jointly.

The BCI parameters used in model selection in the synchronous BCI dataset consisted of the selection of the time segment from which the features are extracted and the number of CSP filters. To adjust the values of these parameters, the system should be customized based on the brain characteristics of each individual subject. To get an acceptable performance with respect to the synchronous datasets, we usually discard some parts of the movement period in each trial. The choice of the time segment from which features are extracted, has a major role in the performance of the learning system. In datasets with many channels we also used CSP to combine different channels. As a result, the number of CSP filters was also included as a parameter in model selection.

In self-paced BCIs, the classification problem is a sequential learning problem. To be able to apply static classifiers to this problem, we used sliding windows with overlap. The size of the window and the overlap of two consecutive windows were parameters in the self-paced datasets.

### Statistical Tests

Instead of using empirical approaches that have been commonly used in the BCI field to compare the performance of different algorithms, the Friedman statistical test is used in this study. The Friedman test [47][48] is a non-parametric statistical test, which ranks different classifiers for each subject separately. Then it averages the ranks over all subjects. The null hypothesis assumes that all algorithms have the same performance so they have the same rank. In other words, it assumes the difference between different algorithms is random. The test statistic has a Chi-square distribution and if the p-value is low enough to reject the null hypothesis, we can conclude that the difference between the algorithms is not random.

If the null hypothesis is rejected, another statistical test is to identify which algorithms are the source of difference. We then conduct the Holm’s test as the post-hoc statistical test. The classifier with the best rank is selected as the control classifier, then a pairwise comparison of all the other classifiers and the control classifier is performed. In this statistical test, the null hypothesis states that the control classifier and the other classifier have the same mean rank. We have divided the algorithms based on the outcome of the Holm’s test into two categories: the recommended and not recommended. The recommended classifiers include the control classifier, i.e., the best one and any other classifier that we are not able to prove it has a worse performance than the best classifier. All the others were deemed to belong to the not recommended category.

## Datasets

Five sensory motor BCI datasets consisting of 29 subjects were used to evaluate different methodologies studied in this paper. These are the datasets I [17], IIa [18] and IIb [19] of the BCI competition IV, and dataset IIIa [20] from BCI competition III and SM2 from [21].

Table 1 shows a summary of each of the datasets. While SM2 and BCICIV1 datasets were used to evaluate different BCI designs in self-paced paradigm, the remaining datasets were used for synchronous BCI systems evaluation.

**Table 1:** Specification of datasets used in this paper.

Dataset	Type	Number of Subjects	Task	Number of Channels
BCICIII3b	Synchronous	3	left hand vs. right hand	2
BCICIV2b	Synchronous	9	left hand vs. right hand	3
BCICIV2a	Synchronous	9	left hand vs. right hand vs. both feet vs. tongue	22
BCICIV1	self-paced	4	left hand, right hand and foot vs. No-Control	59
SM2	self-paced	4	right index finger vs. No-Control	10

Dataset I from competition IV (BCICIV1) was recorded from 4 subjects performing motor imagery tasks (left hand, right hand or foot imagery). Each subject participated in two sessions of brain signal recording. The first session, namely the calibration phase of recording is used for training the BCI system. The second session of signal recording is used for the evaluation of the BCI system. This dataset consists of 59 EEG channels (corresponding to 59 sensors) that were spread around the sensory motor area of the brain. In the calibration phase, each subject was assigned to perform two of the three classes of motor imagery tasks: left hand, right hand, or foot imagery movements. There were 200 trials of imagery movements that were balanced between two classes. Each trial was 8 seconds long, the length of the motor imagery intervals in the evaluation session varied between 1.5 and 8s. The NC intervals were also between 1.5 and 8s. Getting a very good performance on this 3-class self-paced dataset is very challenging, therefore we converted the problem into a binary classification in which the aim is to decide whether an output is a control state or a No-Control state.

The SM2 dataset was collected from 4 subjects attempting to activate a switch by performing a right index finger movement. At random intervals, a cue was displayed for the subjects. The subjects attempted to activate a switch by moving their right index finger after the cue appeared. This dataset is a self-paced data and the EEG was recorded from 10 channels positioned over the supplementary motor area and the primary motor cortex (i.e. FC1-4, FCz, C1-4, Cz). In this dataset, for each subject, an average of 10 sessions of recording of the brain activity was performed for six days. In each session, the period between any two trials varied and the subjects performed actual movement. A detailed description of this dataset is in [21].

The BCICIV1 and SM2 datasets have two challenging properties: 1) since the data were recorded in a self-paced manner in the evaluation set, the classifier does not have any clue about the start time of a movement imagination trial; 2) these datasets contain periods in which the user has no BCI control intentions, which makes the classification problem extremely challenging.

Dataset IIa from the BCI competition IV (BCICIV2a) was recorded for 9 subjects performing 4-class motor imagery (left hand and right hand, both feet and tongue imagery movements) tasks. The data consists of 19 channels along the scalp and the challenge with this dataset is the 4-class classification task. Dataset IIb from BCI competition IV (BCICIV2b) was recorded for 9 subjects performing 2-class motor imagery (left hand and right hand) tasks. The data consists of 3 channels (i.e. C3, CZ and C4) along the motor cortex of the scalp. Dataset IIIb from BCI competition III (BCICIII3b) was recorded from 3 subjects performing 2-class motor imagery (left hand and right hand) tasks. The data has 2 channels (i.e. C3, C4) from the motor cortex area of the brain.

## Results and Discussion

We applied different combinations of feature extraction, classification and model selection methods to five datasets. Tables 2 and 3 show the performance for synchronous and self-paced datasets, respectively, with the best performing feature extraction/classification combinations typed in bold. Qualitative comparison of the different feature extraction/classification combinations suggests the following: 1) for synchronous BCI systems, logistic regression classification outperforms the other classifiers (Table 2). This is regardless of the feature extraction methodology used. 2) For self-paced BCIs, (Table 3), both logistic regression and MLP classifiers yield better performances. In addition in both self-paced and synchronous BCIs Tables 2 and 3 show that for the subjects with the higher numbers of EEG channels, BP outperforms Morlet mainly due to the application of CSP on the channels. In datasets with lower numbers of EEG channels, the use of Morlet features outperforms that of BP features.

**Table 2:** The accuracy of classifiers for synchronous BCI operation for all subjects. For each subject the accuracy on the test data is shown. For each classification algorithm the first column shows the results of BP features and the second column shows the results of Morlet features.

	**Boosting**		**Logistic**		**Random Forest**		**SVM**		**LDA**		**QDA**		**MLP**	
	BP	Morlet	BP	Morlet	BP	Morlet	BP	Morlet	BP	Morlet	BP	Morlet	BP	Morlet
Subject1 (O3)	75.47	80.5	80.5	82.39	74.84	79.25	81.76	77.36	81.13	74.21	79.25	60.38	78.62	**83.65**
Subject2 (S4)	71.11	77.96	70.19	**83.89**	68.52	79.26	71.11	83.52	70	81.11	70.37	72.41	70.37	82.22
Subject3 (X11)	72.22	**78.15**	74.26	**78.15**	71.48	77.78	72.78	77.78	74.07	76.48	74.81	72.22	74.81	76.67
Subject4 (100)	66.23	61.84	60.96	68.86	63.6	65.35	64.04	64.91	61.4	**75.88**	63.16	58.77	63.6	69.3
Subject5 (200)	53.47	51.84	56.33	58.37	54.29	54.29	56.73	**59.18**	56.33	55.92	54.69	55.51	56.33	58.37
Subject6 (300)	56.52	54.35	56.09	53.48	51.74	51.74	51.74	45.22	56.09	49.13	54.35	46.52	**57.83**	51.74
Subject7 (400)	89.25	90.88	94.79	94.79	91.21	92.83	95.11	94.14	93.81	94.46	**95.77**	83.39	92.18	94.46
Subject8 (500)	61.9	86.45	67.77	**91.58**	63.37	85.71	68.13	85.71	65.57	87.18	67.03	76.56	67.4	85.71
Subject9 (600)	74.1	79.68	75.7	82.87	74.1	80.48	65.74	80.88	76.1	82.07	75.3	60.96	76.89	**83.27**
Subject10 (700)	54.31	70.69	53.02	72.84	49.14	**76.29**	52.59	70.69	59.05	71.55	57.33	64.66	51.29	71.12
Subject11 (800)	91.74	83.91	**92.17**	83.48	90	86.52	**92.17**	80.87	**92.17**	80.87	86.52	67.83	90.87	80
Subject12 (900)	78.37	82.45	77.55	**86.12**	75.92	82.86	76.73	**86.12**	77.96	76.33	77.96	68.57	77.14	83.67
Subject13 (1)	79	71.53	79	60.85	**81.85**	73.31	81.14	61.57	74.38	50.89	52.67	26.33	**81.85**	58.01
Subject14 (2)	51.59	53.36	61.13	54.42	53.36	51.24	57.24	57.6	**62.9**	32.86	38.87	25.8	58.3	54.77
Subject15 (3)	78.75	83.15	86.45	**86.81**	78.02	82.78	84.25	78.02	80.22	50.55	41.03	27.47	85.35	83.15
Subject16 (4)	71.49	32.02	**73.68**	41.23	**73.68**	45.18	71.05	36.84	60.96	35.53	36.84	30.26	72.81	34.65
Subject17 (5)	56.16	33.33	**60.14**	40.58	58.33	35.14	56.88	41.67	50	28.26	34.06	28.26	**60.14**	38.77
Subject18 (6)	51.63	24.65	56.74	26.98	52.09	26.05	57.21	25.58	54.42	27.91	33.95	26.98	**59.07**	26.05
Subject19 (7)	84.48	64.98	**87.36**	56.32	81.95	71.48	**87.36**	64.26	70.4	32.49	25.27	31.41	87	60.29
Subject20 (8)	77.12	54.98	80.81	61.62	79.34	63.47	**81.55**	62.36	75.65	37.27	46.49	31	80.44	61.62
Subject21 (9)	78.03	46.97	83.71	39.39	82.95	56.44	84.09	45.08	73.86	41.29	34.47	25	**84.85**	44.32

**Table 3:** The AUC of classifiers for self-paced subjects. For each classification algorithm the first column shows the results of BP features and the second column shows the results of morlet features.

	**Boosting**		**Logistic**		**Random Forest**		**SVM**		**LDA**		**QDA**		**MLP**	
	BP	Morlet	BP	Morlet	BP	Morlet	BP	Morlet	BP	Morlet	BP	Morlet	BP	Morlet
Subject22 (22)	0.46	0.56	**0.64**	0.58	0.49	0.49	0.41	0.48	**0.64**	0.57	0.62	0.55	0.63	0.56
Subject23 (23)	0.54	0.61	0.67	0.68	0.53	0.55	0.5	0.59	0.64	0.68	0.63	0.53	0.66	**0.7**
Subject24 (24)	0.6	0.58	**0.66**	0.58	0.58	0.51	0.6	0.52	0.65	0.58	0.64	0.54	0.63	0.57
Subject25 (25)	0.36	0.31	0.78	0.77	0.6	0.69	0.47	0.6	**0.79**	0.77	0.68	0.66	**0.79**	0.73
Subject26 (a)	0.59	0.53	**0.66**	0.55	0.64	0.53	0.56	0.53	0.65	0.53	0.61	0.5	**0.66**	0.57
Subject27 (b)	0.79	0.77	0.82	**0.83**	0.78	0.81	0.72	0.76	0.66	0.8	0.72	0.73	0.82	**0.83**
Subject28 (f)	0.49	**0.54**	0.51	0.53	0.51	0.53	0.49	0.51	0.5	0.53	0.48	0.51	0.49	0.52
Subject29 (g)	0.52	0.5	0.53	**0.58**	0.52	0.51	0.53	0.51	0.53	0.55	**0.58**	0.52	0.53	**0.58**

The Friedman statistical test was performed to compare the performance of the different classification methods. This was done for both the BP and Morlet feature extraction methods for both types of BCIs (synchronous and self-paced), resulting in four different comparison settings. In Settings 1 and 2, the BP and Morlet features with different classifiers were applied on the synchronous datasets, respectively, and in Settings 3 and 4, the BP and Morlet features with different classifiers were applied on the self-paced datasets, respectively.

The reason we compared the performance of different classifiers when a single feature extraction methodology was used was that the nature of feature spaces was different for each setting and the results could thus be misleading. For example, when we use the BP features, the feature space is of low dimensions compared to the settings where the Morlet feature is used. Therefore, classification methodologies could only be compared when they are applied on the same feature space. Since the classification problem in synchronous and self-paced BCIs was different, it only make sense to compare the classifiers on self-paced and synchronous datasets separately.

The comparison between different classifiers was performed as follows: (a) we ranked each classification algorithm based on its performance on the test data. For example, for subject1 (O3), when the Morlet features were used, the MLP was the best classifier, LR was the second best and so on. We ranked the algorithms for all subjects separately, then we averaged the ranks, (b) the Friedman test was then applied on the resulting ranked results. (c) if the null hypothesis was rejected by the Friedman statistical test, we performed a second set of statistical tests. In particular, we used Holm’s test with the winning algorithm being the control in the test.

The average rank of the different classifiers that are based on the number of wins for the synchronous and the self-paced datasets respectively are given in Table 4. These results show that depending on the feature sets and the dataset type (synchronous versus self-paced), both linear classifiers such as LR and LDA and non-linear ones (e.g., MLP, RF) could be among the top performing classifiers. Another interesting observation is that in all settings, the average rank of the LR classifier is better than LDA. This is due the fact that the data in BCI is noisy and usually have many outliers. As discussed in the previous sections, unlike LR, LDA is sensitive to outliers and is therefore not robust. Comparing the ensemble classifiers shows that RF has a better average rank compared to BST; this is again due the sensitivity of BST to outliers.

**Table 4:** Average Rankings of the classification algorithms for both synchronous and self-paced datasets. The number in the parenthesis corresponds to the average rank of the algorithm among different subjects. For each feature extraction method the classifiers typed in bold are the recommended ones. The recommended classifiers are selected based on the results of the statistical tests.

	**Synchronous**		**Self-paced**	
	**BP** (Setting 1)	**Morlet** (Setting 2)	**BP** (Setting 3)	**Morlet** (Setting 4)
1	**MLP(2.92)**	**LR(2.45)**	**LR(1.81)**	**LR(1.87)**
2	**LR(2.97)**	**RF(3.3)**	**MLP(2.75)**	**MLP(2.56)**
3	**SVM(3.11)**	**MLP(3.42)**	**LDA(3.06)**	**LDA(2.81)**
4	**LDA(4.09)**	**SVM(3.57)**	QDA(4.18)	BST(4.25)
5	BST(4.54)	BST(4.16)	RF(4.87)	RF(4.87)
6	QDA(5.11)	LDA(4.45)	SVM(5.5)	SVM(5.81)
7	RF(5.21)	QDA(6.61)	BST(5.81)	QDA(5.81)

In Setting 1, as shown in Table 4, MLP was the best performing classifier, i.e., when the BP features were used on synchronous datasets. Since the null hypothesis was rejected by the Friedman statistical test (p-value = 1.4e-4), the Holm’s post-hoc was performed and all classifiers were compared to MLP (control classifier). The p-values corresponding to pairwise comparison of classifiers are shown in Table 5. For *α* = 0.1, all hypothesis with p-value less than 0.0333 were rejected. According to the Holm’s test results, there is not significant difference between the performance of MLP, LR, SVM and LDA. Thereby, these classifiers are the recommended ones for the BP features in synchronous data. The other classifiers, i.e., BST, QDA and RF had a poor performance for this type of feature extraction.

**Table 5:** P-values corresponding to pairwise comparison of different classifiers. *α* is chosen to be 0.1. For settings 1 and 2 all hypothesis with p-value less than 0.0333 are rejected. For setting 3 and 4 all hypothesis with p-value less than 0.05 are rejected. The results are rounded up to 4 decimal places.

Setting 1(Synchrounous, BP)		Setting 2(Synchrounous, Morlet)		Setting 3(Self-paced, BP)		Setting 4(Self-paced, Morlet)	
hypothesis	*P*−*value*	hypothesis	*P*−*value*	hypothesis	*P*−*value*	hypothesis	*P*−*value*
RF vs. MLP	0.0006	QDA vs. LR	0.0	SVM vs. LR	0.0002	SVM vs. LR	0.0002
QDA vs. MLP	0.0010	LDA vs. LR	0.0026	BST vs. LR	0.0006	QDA vs. LR	0.0002
BST vs. MLP	0.0151	BST vs. LR	0.0101	RF vs. LR	0.0045	RF vs. LR	0.0054
LDA vs. MLP	0.0801	SVM vs. LR	0.0932	QDA vs. LR	0.0278	BST vs. LR	0.0278
SVM vs. MLP	0.7750	MLP vs. LR	0.1431	LDA vs. LR	0.2471	LDA vs. LR	0.3854
LR vs. MLP	0.9430	RF vs. LR	0.1985	MLP vs. LR	0.3854	MLP vs. LR	0.5244

In Setting 2, the best performing classifier was LR (Table 4). The p-value for the Friedman test was 1.7e-8; therefore, the difference between the classifiers is not random. The Holm’s test suggests that RF, MLP, and SVM are as good as the LR classifier (Table 5 setting 2).

In Settings 3 and 4, the LR classifier performed better than others (Table 4). In Setting 3 the Friedman’s test p-value was 7.16e-4, and in Setting 4 Friedman’s test p-value was 1.8e-4. The Holm’s test results suggested that there was not significant difference between MLP, LDA and LR (Table 5 setting 3 and 4). All hypothesis with p-value less than 0.05 were rejected.

Among the classifiers used in this study, RF, BST and MLP are inherently designed to handle multi-class classification and the others (i.e., SVM, LR, LDA and QDA) are used in a one against others setting to handle multi-task problems. Therefore, in addition to the four settings discussed above, we have also performed two other statistical tests. The aim was to determine which classifiers yield the best results in binary-task BCIs and which one(s) yield the best results in multi-task BCIs. From the total of 21 subjects in the synchronous BCIs datasets, 12 had performed binary tasks and 9 had performed multi-task control of BCIs. Therefore, we performed separate statistical tests for binary and for multi-task datasets. The average rank of different classifiers for the binary and for the multi-task datasets are given in Table 6. Table 6 shows that in binary-task BCIs for both BP and Morlet features the best performing classifier is an inherently binary classifier (i.e., SVM in binary-task BCIs with BP features and LR in binary-task BCIs with Morlet features). Furthermore, in multi-task BCIs for both kinds of features the best performing classifier is an inherently multi-class classifier (i.e. MLP in multi-task BCIs with BP features and RF in multi-task BCIs with Morlet features).

**Table 6:** Average Rankings of the classification algorithms for binary and multi-class classification in synchronous datasets. The number of subjects in binary task was 12 and the number of subjects in multi-task BCIs was 9. The number in the parenthesis corresponds to the average rank of the algorithm among different subjects. For each feature extraction method the classifiers typed in bold are the recommended ones. The recommended classifiers are selected based on the results of the statistical tests.

	**BP**		**Morlet**	
	**Binary**	**Multiclass**	**Binary**	**Multiclass**
1	**SVM(3.33)**	**MLP(2.11)**	**LR(1.87)**	**RF(2.50)**
2	**LDA(3.41)**	**LR(2.22)**	**MLP(3.20)**	**SVM(3.00)**
3	**MLP(3.54)**	**SVM(2.83)**	RF(3.91)	**LR(3.22)**
4	**LR(3.54)**	**RF(3.88)**	LDA(3.91)	**MLP(3.72)**
5	**QDA(3.70)**	BST(4.94)	SVM(4.0)	**BST(3.94)**
6	**BST(4.25)**	LDA(5.0)	BST(4.33)	LDA(5.16)
7	RF(6.20)	QDA(7.0)	QDA(6.74)	QDA(6.44)

For each of the groups of the subjects, we performed the Friedman test and the Holm’s post-hoc test. This is done to statistically compare the performance of other classifiers with respect to the best performing classifier in each case. The p-values corresponding to pairwise comparison of classifiers are given in Table 7. Table 7 suggests that for the BP features, MLP, LR and SVM are recommended in both the binary and multi-class BCIs. According to Table 4, these classifiers are also recommended in Setting 1 (corresponding to 21 subjects performing synchronous BCIs with BP feature extraction method). Table 7 also suggests that, for Morlet features, LR and MLP classifiers are recommended for both the binary and multi-class BCIs. According to Table 4, these classifiers are among the recommended ones in Setting 2 (corresponding to 21 subjects performing synchronous BCIs with Morlet feature extraction method). The results suggest that among the classifiers that are designed to handle multi-task classification, RF and MLP are recommended to be used in multi-task BCIs. The other observation is that even in multi-task BCIs some inherently binary classifiers are among the recommended classifiers. The results of Table 4 are, however, more reliable as the number of subjects in Settings 1 and 2 is almost twice the number of subjects considered in Table 6.

**Table 7:** P-values corresponding to pairwise comparison of different classifiers. *α* is chosen to be 0.1. For binary task BCIs with BP features all hypothesis with p-value less than 0.02 are rejected. For multi-task BCIs with BP features all hypothesis with p-value less than 0.0333 are rejected. For binary task BCIs with Morlet features all hypothesis with p-value less than 0.1 are rejected. For multi-task BCIs with Morlet features all hypothesis with p-value less than 0.025 are rejected. The results are rounded up to 4 decimal places.

**(Binary, BP)**		**(Multi-task, BP)**		**(Binary, Morlet)**		**(Multi-task, Morlet)**	
hypothesis	*P*−*value*	hypothesis	*P*−*value*	hypothesis	*P*−*value*	hypothesis	*P*−*value*
RF vs. SVM	0.0011	QDA vs. MLP	0.0	QDA vs. LR	0.0	QDA vs. RF	0.0001
BST vs. SVM	0.2986	LDA vs. MLP	0.0045	BST vs. LR	0053	LDA vs. RF	0.0088
QDA vs. SVM	0.6706	BST vs. MLP	0.0053	SVM vs. LR	0.0159	BST vs. RF	0.1560
LR vs. SVM	0.8132	RF vs. MLP	0.0808	RF vs. LR	0.0206	MLP vs. RF	0.2300
MLP vs. SVM	0.8132	SVM vs. MLP	0.4781	LDA vs. LR	0.0206	LR vs. RF	0.4781
LDA vs. SVM	0.9247	LR vs. MLP	0.9131	MLP vs. LR	0.1305	SVM vs. RF	0.6234

## Conclusion

We have built the first general open source Python-based framework to compare the performance of different algorithms in BCIs. Using this framework, we performed a comprehensive comparison between 14 different BCI designs (two feature extraction methods and seven classification methods for each feature extraction methodology) over 29 sensory motor BCI subjects in synchronous and self-paced BCI paradigms.

Our results show that the Logistic Regression (LR) and Multi-Layer Perceptron (MLP) classifiers are among the best performing classifiers and are recommended in all different designs. LR is linear like Linear Discriminant Analysis (LDA) while MLP is a very powerful nonlinear classifier. Both the LR and MLP classifiers are prone to over-fitting; however, in both cases we have included regularization terms to avoid overfitting. The observation that LR was among the best classifiers suggested that the feature space of our task was somewhat linearly separable.

Unlike most publications in the BCI field which recommend the LDA as the best classifier, our findings show that for each feature extraction method at least the recommended classifiers should be tested and then the best classifier should be selected based on the cross-validation results. In general, there is not a best classifier or best feature extraction method that outperforms all others. For each subject, the combination of classifier, feature and model parameters should be tuned together, and finally the method with the best performance on the training data set should be selected as the final model for testing on unseen data.

Finally, we should emphasize that classification is just one step in our framework, and to get acceptable performance other steps are also important. Pre-processing of the data, feature extraction, and feature selection all change the distribution of the data in the feature space and have a major role in getting good results. Therefore, a BCI system should be viewed as a unit consisting of different blocks in which all the block settings and parameters should be adjusted jointly for each individual subject.

## Appendix

### 1

All implementations are performed in Python. For classification algorithms Scikit-learn toolbox [49] has been used. For MLP classifier, Theano toolbox [50][51] has been used. The source code of our framework and the SM2 dataset are both available at https://github.com/hosseinbs/BCI-Comparison-Framework.

### 2

For model selection different set of parameters have been evaluated. The set of parameters evaluated for all classifiers (i.e. classifier parameters) are given in Table 8. All these parameters along with the BCI parameters were optimized in the training phase.

**Table 8:** List of classifier parameters tuned in the training phase.

Random Forest	Number of trees
	Maximum number features evaluated to split each node
	Maximum depth of each tree
	Minimum number of samples in each leaf
SVM	C
	Gamma
LR	Regularization type
	Regularizer coefficient
Boosting	Number of trees
	Maximum number features evaluated to split each node
	Maximum depth of each tree
	Learning rate
MLP	Number of neurons in hidden layer
	L1 coefficient
	L2 coefficient
	Learning rate
GDA	None
